# Duplication in *ECR* near *HMX1* and a SNP in *GATA6* Genes Regulate Microtia in Awassi Sheep

**DOI:** 10.3390/genes11060597

**Published:** 2020-05-28

**Authors:** Khaleel I. Z. Jawasreh, Haitham Daif-Allah Al-Omari

**Affiliations:** Department of Animal Production, Faculty of Agriculture, Jordan University of Science and Technology (JUST), Irbid 22110, Jordan; hdahoaao94@gmail.com

**Keywords:** anotia, modifier gene, pinna, earless

## Abstract

Microtia and anotia are hereditary traits characterized by an underdevelopment or complete absence of the outer ear. These congenital malformations observed in many species can exist as part of various syndromes or as an isolated trait as seen in the fat-tailed Awassi sheep breed. Our study aims to identify the genetic mutations causing microtia in Awassi sheep by DNA sequencing. DNA was extracted from blood samples randomly collected from 84 Awassi sheep (16 earless, 41 short ear and 27 normal ear) across different farms. *GATA6* exons 1, 2, 4, 6 and 7, CLRN1 intron 3, DCC intron 2, ECR near *HMX1* and the intergenic region between *GATA6* and MIB1 genes were screened, amplified and sequenced. Allele and genotype frequencies were calculated by direct counting. Association was performed using chi-squared test for goodness-of-fit. Results showed mutations in only two genes significantly associated with microtia in Awassi: duplication in part of ECR near *HMX1* (6:114293121-6:114293196) and a SNP at *GATA6* exon 7 (23:34498242). Association results revealed that the ECR locus accounts for the microtia phenotype, while *GATA6* exon 7 acts as a modifier gene. Genetic screening for these loci can be used to improve selection against microtia in Awassi sheep.

## 1. Introduction

Sheep (*Ovis aries*) are even-toed ruminants domesticated and raised mainly as a source for meat, wool and milk. For sheep—as other animals—the ear is an important auditory organ for sound and environment perception; the outer ear is evolutionary-shaped as a funnel that collects and amplifies external sound stimuli, converted afterwards into nerve impulses and sent to the central nervous system for interpretation and sensorial perception. Furthermore, the mammalian ear plays additional functions including heat dissipation and body balance [1].

Microtia is a congenital ear malformation characterized by the under-development of the outer ear, while anotia is its complete absence. These conditions can exist as isolated traits or as part of various syndromes and occur in many species including humans, mice and various livestock species. Although cases of anotia or microtia in humans are usually associated with some facial abnormalities, kidney and heart problems and vertebral deformities [2], investigations on the physiological and health repercussions of this malformation in domestic animals are scarce. In sheep, microtia is generally observed as an isolated condition. The Awassi, a fat-tailed sheep from southwest Asia, is among breeds with isolated microtia, with no apparent association with other defects and disorders [3]. However, this condition often reduces the animal’s market value. Moreover, available reports on the etiology and inheritance of microtia in different sheep breeds are controversial. Using GWAS or local sequence alignment techniques, microtia was reported to be associated with mutations in *GATA6* (GATA binding protein 6) in Awassi sheep [3], DCC (deleted in colorectal carcinoma) in Duolang sheep [4], *CLRN1* (Clarin 1) in Valle del Belice [5] and a duplication within an evolutionary conserved region (*ECR*) near *HMX1* (H6 family home-box 1) in Altay sheep [6]. Following these debatable reports in the underlying genetic causes behind the microtia phenotype in different sheep breeds, this study aims at identifying the possible mutation(s) behind the inheritance of microtia in the Awassi breed.

## 2. Materials and Methods

### 2.1. Ethical Approval

All experimental animal protocols were approved by the Animal Care and Use Committee (ACUC) at Jordan University and Science Technology (JUST), approval number 576/3/3/16.

### 2.2. Animal and Sample Collection

**Figure 1:**
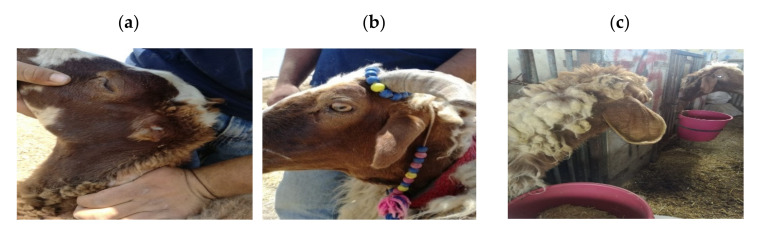
Ear types of Awassi sheep. (**a**) Earless, (**b**) short and (**c**) normal.

The study was conducted at JUST, department of Animal Production–Animals Biotechnology laboratory, Jordan. A total of 84 Awassi sheep (16 earless, 41 short ear and 27 normal ears) were sampled from different farms across the Northern part of Jordan (Figure 1). The animals were unrelated and of different ages and sex. Approximately 4-mL blood samples were collected from the jugular vein using EDTA blood collection tubes and stored at −20 °C until lab analysis. In parallel, breeders were interviewed to gauge their opinion regarding the association between the general performance and ear size variation within sheep.

### 2.3. Genomic DNA Extraction

DNA was extracted from the collected blood using nuPREP Blood DNA Mini Kit (Analytic Jene AG, Jena, Germany) according to manufacturer’s instructions. DNA quality was tested using 1.5% agarose gel electrophoresis solved in 1X TBE buffer and visualized using Ultra Violet imager (Alpha Imager HP 220 V, San Jose, CA, USA).

### 2.4. Polymerase Chain Reaction (PCR)

The following genes were amplified using PCR (Life Pro Thermal Cycler, Hangzhou, China): *GATA6* exon 1, 2, 4, 6, 7; *CLRN1* intron 3, *DCC* intron 2, part of *ECR* near *HMX1* and part of intergenic region between *GATA6* and *MIB1*. All primers were designed using Primer 3 Plus [7] and the optimal annealing temperatures were determined using gradual thermal gradient PCR. PCR reactions were set up using 100 to 200 ng/µL genomic DNA, 10 µM of each forward and reverse primer and 15 µL One Taq^®^ 2× Master Mix with Stander Buffer DNA polymerase, and nuclease free water use to reach a final volume of 25 µL. The following thermal cycling conditions of 30-s denaturation at 94 °C followed by 35 cycles of 30 s at 94 °C, then a 50-s annealing at 57–64 °C and a 1-min extension at 68 °C with a final elongation at 68 °C for 5 min (Table 1) were used. PCR product-quality was tested using 1X TBE 1.5% agarose gel electrophoresis.

### 2.5. Sequencing Procedure

The PCR products were sequenced in both directions at Princess Haya Biotechnology Center (PHBC, AR-Ramtha, Jordan) of JUST University, using the Sanger sequencing method following DNA purification and preparation. Mutations were detected using multiple sequence alignment in Bioedit software [8].

### 2.6. Statistical Analysis

Allele and genotype frequencies were calculated by direct counting. A chi-squared test for goodness-of-fit was performed. Significant differences were determined using SAS [9]. Interaction effect between the mutations was calculated directly after combining the genotypes as 3 × 3 interactions for the *ECR* near *HMXI* and exon 7 of the *GATA6* genes mutations and tested by chi-squared.

## 3. Results

### 3.1. GATA6 Gene Exons 1, 2, 4 and 6

Amplification of exons 1, 2 and 4 of *GATA6* and surrounding parts (parts of introns around the exons) resulted in PCR products of lengths: 541, 247 and 281 bp, respectively. A T to C mutation was observed at position 23:34503270 of the intron 1. Another SNP (C/T) was detected in exon 2 at genomic locus 23:34502143. In exon 4, two missense mutations (G/A) were observed. The first mutation at locus 23:34499932 was responsible for the substitution of Glutamine by Lysine. The second mutation at locus 23:34499918 caused the substitution of Arginine by Tryptophan. Amplification of exon 6 of *GATA6* resulted in a 365 bp fragment. Sequencing revealed C to A missense mutation at locus 23:34499639 (rs405842265) substituting Alanine to serine (Gene and genotype frequencies are illustrated in Table S1). However, all of these observed mutations were not significantly associated (*p* < 0.05) with ear phenotype variation in Awassi sheep (Table 2).

### 3.2. GATA6 Gene Exon 7

**Figure 2:**
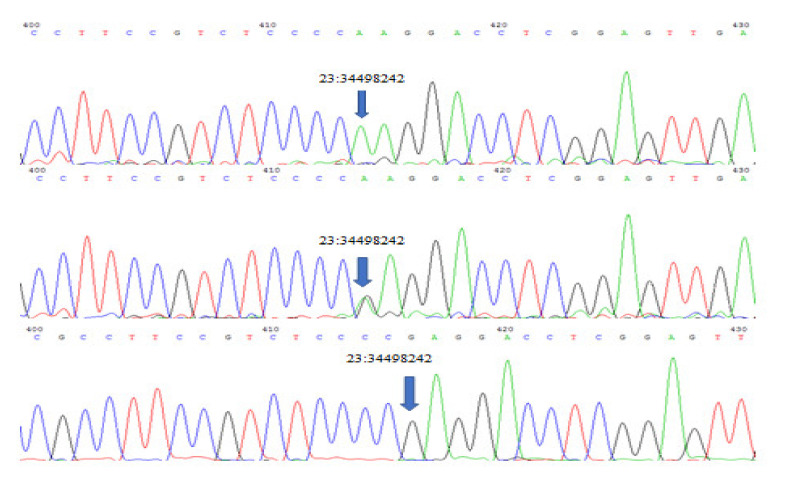
Three genotypes were detected at genomic locus: 23:34498242, 5606 bp (relative to the gene size) in *GATA6* Exon 7 GA, AA and GG.

Sequencing of *GATA6* exon 7 revealed a significant (*p* < 0.01) novel A to G missense mutation on position 23:34498242 which replaces tryptophan with glycine. Three genotypes (AA, GA and GG) (Figure 2) were detected for this mutation with significant (*p* >0.01) differences between each phenotype. Population genetic analysis showed AA genotype to be frequently found in all ear phenotypes, especially in earless, while GA and GG were only found in the short and normal-ear phenotypes (Table 3).

### 3.3. Intergenic Region between GATA6 and MIB1 Genes

Amplification of the intergenic region between the *GATA6* and MIB1 genes resulted in 380 bp and 367 bp (same as wild type) fragments (Gene and genotype frequencies are illustrated in Table S2). No significant associations between product length, genotypes and ear phenotypes were detected (*p* < 0.05).

### 3.4. CLRN1 and DCC Genes

Amplification part of CLRN1 intron 3 and DCC intron 2 resulted in 769 and 699 bp fragments, respectively. CLRN1 intron 3 revealed a statistically insignificant mutation (*p* < 0.05) at 1:235105286 (rs419889303) with the GG genotype present in 100% of all normal-ear samples and 70% in the short ear and earless samples, while GA and AA had low genotypes frequencies in both earless and microtia samples (GA: 20%, AA: 10%).

DCC intron 2 showed a statistically insignificant (*p* > 0.05) G-to-A mutation (rs402740419). GG (80% in Earless, 60% in Short and 100% in Normal) appeared at high frequencies in all phenotypes. AA was present in 20% of earless, while GA in 40% of short-ear samples.

### 3.5. ECR Near HMX1 Gene

**Figure 3:**
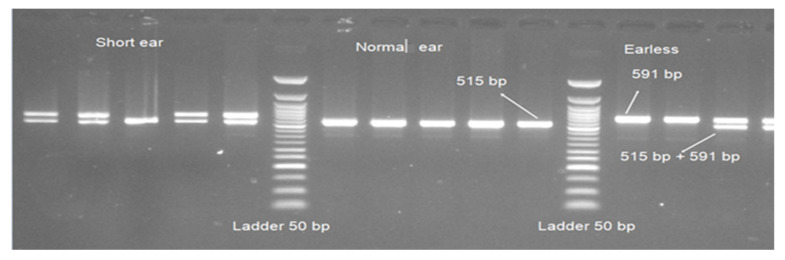
Polymerase chain reaction (PCR) products of ECR near *HMX1* gene and Ladder 50 bp in 1.5% agarose gel.

The amplification of evolutionarily conserved region (*ECR*) near homeodomain transcription factor *1(HMX1)* gene resulted in 591 and 515 bp fragments (Figure 3). All 27 normal Awassi samples had a product size of 515 bp. After amplification, 39 out of 41 short-ear samples had two bands (of product size 591 and 515 bp) and two samples had bands of only 515 bp in size. From the 16 earless samples amplified, 6 had a product size of 591 bp and 10 samples had both 591 and 515 bp.

The PCR product size of ECR near *HMX1* (duplication of 76 bp) showed a highly significant association with ear size in Awassi sheep (*p* < 0.0002), in addition to the significant difference (*p* < 0.001) between affected animals (earless versus short ear) (Table 3).

### 3.6. Interaction and Association between Mutations at GATA6 Exon 7 and ECR near HMX1 and Microtia Phenotype in Awassi Sheep

The interaction between the missense mutation found in exon 7 of the *GATA6* gene (locus 23:34498242) and the duplication mutation in ECR near *HMX1* gene resulted in only seven genotypes in the sampled population: DDAA, DdAA, DdAG, DdGG, ddAA, ddAG and ddGG (D: duplication mutation on *ECR–HMX1*, G: Guanine and A: Adenine). Most of the short-ear animals (39/41) presented the Dd genotype irrespective of GATA 6 gene genotypes (AA, AG or GG), where 5% were of ddGG, 62.5% of earless animals were DdAA and the remainder (37.55%) were DDAA. These genotype ratios indicate that the presence of the mutated G allele in exon 7 of *GATA6* gene may prevent the appearance of the earless phenotype if it is associated with DD or Dd genotypes. The results showed that all normal-ear animals carried the dd (ECR–*HMX1*) genotype regardless of *GATA6* (Exon 7) genotype variations (Table 3).

## 4. Discussion

Jawasreh et al. [3] reported that mutations in the *GATA6* gene may be behind the microtia phenotype in Awassi sheep and Alexandrovich et al. [10] reported *GATA6* to have a role in regulating chondrogenesis. The NCBI database accounts for five major exons (1, 2, 4, 6 and 7) in the *GATA6* gene selected according to their size and high mutation rate. Our study did not find any significant mutations in exon 1 associated with ear size. Sequencing results showed a SNP intron 1 (23:34503270) on chromosome 23 [11]. Although exon 1 did not show any substantial mutations, a synonymous C to T mutation (locus 23:34502143) was found on exon 2 and a missense C to A mutation on exon 6 (23:34499639) [12]. Two missense point mutations were discovered in exon 4, where one was a novel SNP (23:34499932), not previously registered at the international database, while the second (23:34499918) was reported by McLaren et al [13]. Exon 6 also showed a missense mutation of C to A at locus 23:34499639 [12]. While the previous mutations were not significantly associated with ear size, we found a novel SNP in *GATA6* exon 7 (23:34498242) that was significantly associated with ear size variation in Awassi sheep. This point missense mutation was not registered previously and may play an incomplete role (as a modifier mutation) in outer ear malformation in this breed. Jawasreh et al. [3] reported similar results of a SNP at genomic locus 23:34647499 through a genome wide association study. In addition, our study found a deletion mutation (-13 bp) near this locus, not reported in gene databases, and without any significant association with microtia in Awassi sheep.

While Duolang sheep demonstrate continuous variations in ear size due to a SNP (rs402740419) in the intronic region of DCC gene which plays a critical role in ear development [4], Awassi sheep exhibit three distinct ear phenotypes: earless, short and normal. Our sequencing did establish a SNP at the same locus, but without any significant association with microtia. Previously Keino et al. [14] reported the DCC gene to encode a netrin receptor, associated with the alteration from proliferation to terminal development in different tissues. This disagreement in the results may be due to differences in breed genetic backgrounds and the nature of the trait.

Another SNP (rs419889303) was observed within CLRN1 gene on chromosome 1 which has been implicated with microtia in Valle del Belice sheep [5], GG genotype frequencies were 75% in control and 5% in microtia and found to be 100% in control and 70% in both anotia-microtia, GA were 25% in control and 55% in microtia, while in our results, they were 0% in control and 20% in both anotia-microtia samples. AA frequencies were similar to the control (0%), but different for microtia samples (40% versus 10%, respectively). This disagreement may also be due to breed differences. Geller et al. [15] found CLRN1 to be expressed in multiple areas of the body, including hair cells (sensory cells in the inner ear that facilitate sound and motion signals transition to the central nervous system).

A duplication of 76 bp within the intergenic region between *HMX1* and *CZP* (Carboxy peptidase Z) genes was reported in Altay sheep microtia [6] and crop ears in Highland cattle [16]. Researchers suggested that microtia is dominantly inherited in Altay sheep, but without any distinction between different ear types (short and earless) [6]. According to Schorderet et al. [17], mutation on *HMX1* gene cause malformation in the Human external ears. Amplification of this region in Awassi sheep using the same primers [6] generated very similar results with some exceptions. Mutations in this region did not show the earless phenotype in Altay sheep while it was present in Awassi sheep. Although their results suggested a perfect association between the duplication mutation in ECR near *HMX1* and microtia, our results found two microtia samples without this mutation. The study also failed to connect *GATA6* gene in microtia in Altay sheep, while our study established an interaction between this duplication mutation and the novel SNP found in exon 7 of *GATA6* gene in Awassi sheep.

The interaction effect between the microtia phenotype Awassi sheep (our findings) and the duplication mutation ECR near *HMX1* gene strongly suggests that the G allele of the *GATA6* exon 7 mutation contributes to the ear elongation as a modifier mutation (Table 3). Our results suggest that the co-presence of the G allele in the *GATA6* mutation and the Dd genotype of ECR near *HMX1* may contribute to ear length; the G allele of the *GATA6* mutation was not present in the earless phenotype, even those of Dd ECR near *HMX1* genotype; suggesting a contribution of the G allele to ear elongation as it appeared only in the short and long ear animals. The A to G mutation in *GATA6* exon seven was only present in normal (long) and short-ear phenotypes and could be the reason for the large differences and variations in ear types from large pinna in normal Awassi sheep to short (including the two short ears that were of ddGG) and to very small pinna in different sheep breeds found in NCBI (Texel sheep). This mutation replaces tryptophan by glycine and may cause defects in cartilage formation [18]. All previous studies in this field have determined mutations in different genomic loci that cause microtia in sheep within non-coding regions (intronic or intergenic regions), but, for the first time, we have identified a genetic locus associated with ear shortness within the exonic region that directly regulates amino acid translation.

Italian breeders suggested that Valle del Belice sheep with short ear tend to produce more milk [5]. No positive feedback was reported by the Awassi sheep breeders during the brief interviews, where they claimed that fixing ear tags on short ear or earless sheep was problematic and their performance was weaker compared to their normal peers due to nervousness and excessive fear due to unknown reasons. It would be interesting to conduct further associative studies to understand the wider effect of this mutation on the overall health or performance of the Awassi breed.

## 5. Conclusions

A duplication mutation in ECR near *HMX1* modified by a SNP in *GATA6* exon seven plays a significant role in Awassi sheep microtia. Additional investigations based on sequencing of candidate genomic loci in several sheep breeds showing phenotype would help determine a common genetic cause behind microtia in various sheep breeds and improve selection methods against microtia or the rate of its expression in Awassi sheep.

## Figures and Tables

**Table 1:** Forward and reverse primers, product length and optimal annealing temperature for the genes screened in the study.

Gene/Region	Primers	Product Length (bp)	Annealing Temperature (°C)
*GATA6* Exon 1	F: CGCGCTTTTGATAAGTTCTTT R: GGTCGAGGTCAGCGAAGA	541	57
*GATA6* Exon 2	F: AGCGCCTACTCGCCCTAC R: GCACCCACACCAGGACAG	247	64
*GATA6* Exon 4	F: ACCTGGTGTGTCCACCTCTC R: TGGAAGCTCTCTCACCAACA	281	57
*GATA6* Exon 6	F: AGGGAATGGGAGCTGTAGAT R: CAAGGACACTGAAGTTAAACAAGG	365	58.5
*GATA6* Exon 7	F: TATGTCCCGGGGTATTTACG R: CACTCACCACGCGCTTCT	549	57.5
*CLRN1* Intron 3	F: CTGGGTTTCCATGCTCTGAT R: GGCAGGAGTGAACAGGAAAA	769	61
*DCC* Intron 2	F: CGAGGAATAAAATGGAGTCACC R: CAGATGGAACTTGGAAGCAA	699	61
Intergenic region between *MIB1* and *GATA6*	F: CTGGTGGTCCAGTGGTTAAGA R: TGCAGAATTCATCACTGCAAG	367–380	59
*ECR* near *HMX1*	F: GGATGGGGCGAGATAAAGT R: CAGCCACCCTCTCTCTCTTG	515–591	62.5

**Table 2:** Summary of detected mutations within targeted genomic loci.

Gene/Region.	Mutation Locus	Mutant Nucleotides	Amino Acid Alteration	Chi-Squared *p*-Value
*GATA6* Intron 1	23:34503270	T/C	Intronic variant	0.6834
*GATA6* Exon 2	23:34502143	C/T	Synonymous variant	0.7691
*GATA6* Exon 4 (a)	23:34499932	G/A	Glutamine to lysine	0.2865
*GATA6* Exon 4 (b)	23:34499918	G/A	Arginine to tryptophan	0.6092
*GATA6* Exon 6	23:34499639	C/A	Alanine to Serine	0.3074
*GATA6* Exon 7	23:34498242	A/G	Tryptophan to glycine	0.0015
*DCC* Intron 2	rs402740419	G/A	Intronic variant	0.4647
*CLRN1* Intron 3	rs419889303	G/A	Intronic variant	0.1648
Intergenic region between *GATA6* and *MIB1* genes	23:34647527–23:34647539	Deletion of 13 bp	Intergenic variant	0.0880
*ECR nearHMX1* gene	6:114293121–6:114293196	Duplication of 76 bp	Intergenic variant	< 0.0001

**Table 3:** Interaction between mutation at *GATA6* gene Exon 7 ((wild type AA), AG and GG) and evolutionary conserved region (*ECR*) near to *HMX1* gene product size (DD: 591 bp, Dd: 515 +591 bp and dd: 515 bp (wild type)) and its association with ear types in Awassi sheep.

Genotypes		Phenotypes Number (Percentage)			
*ECR* near *HMX1*	*GATA6* Exon 7	Earless	Short Ear	Normal Ear	Chi-Squared *p*-Value
DD	AA	6 (37.5%)	0	0	<0.0001
DD	AG	0	0	0	
DD	GG	0	0	0	
Dd	AA	10 (62.5%)	15 (36.5%)	0	
Dd	AG	0	5 (12.5%)	0	
Dd	GG	0	19 (46.3%)	0	
dd	AA	0	0	13 (48.2%)	
dd	AG	0	0	5 (18.5%)	
dd	GG	0	2 (5%)	9 (33.3%)	

## Supplementary Materials

The following are available online at https://www.mdpi.com/2073-4425/11/6/597/s1, Table S1: GATA6-Exon 6, detected mutation (rs405842265), genotype frequency, allele frequency and Chi-square value for CC, AC and AA genotypes detected in each ear phenotype in Awassi sheep, Table S2, Gene and genotype frequencies and the relationship between ear phenotypes and deletion nucleotides detected in the inter genic region between GATA6 and MIB1 genes in Awassi sheep.

## References

[1] Webster D.B. (1966). Ear Structure and Function in Modern Mammals. Am. Zool..

[2] Luquetti D.V., Heike C.L., Hing A.V., Cunningham M.L., Cox T.C. (2011). Microtia: Epidemiology and genetics. Am. J. Med. Genet. Part A.

[3] Jawasreh K., Boettcher P., Stella A. (2016). Genome-wide association scan suggests basis for microtia in Awassi sheep. Anim. Genet..

[4] Gao L., Xu S.-S., Yang J.-Q., Shen M., Li M.-H. (2018). Genome-wide association study reveals novel genes for the ear size in sheep (Ovis aries ). Anim. Genet..

[5] Mastrangelo S., Sottile G., Sutera A.M., Di Gerlando R., Tolone M., Moscarelli A., Sardina M.T., Portolano B. (2018). Genome-wide association study reveals the locus responsible for microtia in Valle del Belice sheep breed. Anim. Genet..

[6] He S., Zhang Z., Sun Y., Ren T., Li W., Zhou X., Michal J.J., Jiang Z., Liu M. (2019). Genome-wide association study shows that microtia in Altay sheep is caused by a 76 bp duplication of HMX1. Anim. Genet..

[7] Untergasser A., Cutcutache I., Koressaar T., Ye J., Faircloth B.C., Remm M., Rozen S.G. (2012). Primer3--new capabilities and interfaces. Nucleic Acids Res..

[8] Hall T.A. (1999). BioEdit: A user-friendly biological sequence alignment editor and analysis program for Windows 95/98/NT. Nucl. Acids Symp..

[9] Brereton R. (1986). SAS (statistical analysis system). Chemom. Intell. Lab. Syst..

[10] Alexandrovich A., Qureishi A., Coudert A., Zhang L., Grigoriadis A.E., Shah A., Brewer A.C., Pizzey J. (2008). A role for *GATA-6* in vertebrate chondrogenesis. Dev. Boil..

[11] Spooner W., McLaren W., Slidel T., Finch D.K., Butler R., Campbell J., Eghobamien L., Rider D., Kiefer C., Robinson M.J. (2018). Haplosaurus computes protein haplotypes for use in precision drug design. Nat. Commun..

[12] E Hunt S., McLaren W., Gil L., Thormann A., Schuilenburg H., Sheppard D., Parton A., Armean I., Trevanion S.J., Flicek P. (2018). Ensembl variation resources. Database.

[13] McLaren W., Gil L., Hunt S.E., Riat H.S., Ritchie G.R.S., Thormann A., Flicek P., Cunningham F. (2016). The Ensembl Variant Effect Predictor. Genome Boil..

[14] Keino-Masu K., Masu M., Hinck L., Leonardo E., Chan S.S.-Y., Culotti J., Tessier-Lavigne M. (1996). Deleted in Colorectal Cancer (DCC) Encodes a Netrin Receptor. Cell.

[15] Geller S.F., Guerin K.I., Visel M., Pham A., Lee E.S., Dror A.A., Avraham K.B., Hayashi T., Ray C.A., Reh T.A. (2009). CLRN1 Is Nonessential in the Mouse Retina but Is Required for Cochlear Hair Cell Development. PLoS Genet..

[16] Koch C.T., Bruggmann R., Tetens J., Drögemüller C. (2013). A Non-Coding Genomic Duplication at the HMX1 Locus Is Associated with Crop Ears in Highland Cattle. PLOS ONE.

[17] Schorderet D., Nichini O., Boisset G., Polok B., Tiab L., Mayeur H., Raji B., De La Houssaye G., Abitbol M.M., Munier F.L. (2008). Mutation in the Human Homeobox Gene NKX5-3 Causes an Oculo-Auricular Syndrome. Am. J. Hum. Genet..

[18] Lugo P.D.P., Lupiáñez J.A., Meléndez-Hevia E. (2018). High glycine concentration increases collagen synthesis by articular chondrocytes in vitro: Acute glycine deficiency could be an important cause of osteoarthritis. Amino Acids.

