# Supplementary material for: Duplication in ECR near HMX1 and a SNP in GATA6 Genes Regulate Microtia in Awassi Sheep
Source: Genes (Basel). 2020 May 28;11(6):597. doi: 10.3390/genes11060597 (PMC7349607; doi:10.3390/genes11060597)
Supplement: Supplementary file 1 [file genes-11-00597-s001.pdf]

Table S1. GATA6-Exon 6, detected mutation (rs405842265), genotype frequency, allele frequency and Chi-square value for CC, AC and AA genotypes detected in each ear phenotype in Awassi sheep.

	**Allele Frequency**		**Genotype Frequency**			**Chi Square *p*-value**
Phenotype	C	A	CC	AC	AA	
Earless	87.5%	12.5%	75%	25%	0%	
Short Ear	80%	20%	70%	20%	10%	
Normal Ear	90%	10%	80%	20%	0%	0.3074 n.s

n.s: non-significant.

Table 2. Gene and genotype frequencies and the relationship between ear phenotypes and deletion nucleotides detected in the inter genic region between GATA6 and MIB1 genes in Awassi sheep.

**Phenotype**	**SS**	**RR**	**SR**	**Chi Square *p*-value**
Earless	25%	56.25%	18.75%	
Short ear	5%	70%	25%	0.0880 n.s
Normal ear	0%	80%	20%	

n.s: non-significant.
